# Altered functional connectivity of the right caudate nucleus in chronic migraine: a resting-state fMRI study

**DOI:** 10.1186/s10194-022-01506-9

**Published:** 2022-12-02

**Authors:** Ziyu Yuan, Wei Wang, Xueyan Zhang, Xiaoyan Bai, Hefei Tang, Yanliang Mei, Peng Zhang, Dong Qiu, Xue Zhang, Yaqing Zhang, Xueying Yu, Binbin Sui, Yonggang Wang

**Affiliations:** 1 Headache Center, Department of Neurology, Beijing Tiantan Hospital, Capital Medical University, No.119 South Fourth Ring West Road, Fengtai District, 100070 Beijing, China; 2 Tiantan Neuroimaging Center of Excellence, National Clinical Research Center for Neurological Diseases, No.119 South Fourth Ring West Road, Fengtai District, 100070 Beijing, China; 3 Department of Radiology, Beijing Tiantan Hospital, Beijing Neurosurgical Institute, Capital Medical University, No.119 South Fourth Ring West Road, Fengtai District, 100070 Beijing, China; 4 Department of Neurology, The First Affiliated Hospital of Zhengzhou University, No.1, Jianshe East Road, 450000 Zhengzhou, China

**Keywords:** Chronic migraine, Caudate nucleus, Medication overuse headache, Functional connectivity

## Abstract

**Background:**

The definitive pathogenic mechanisms underlying chronic migraine (CM) remain unclear. Mounting evidence from functional and structural magnetic resonance imaging (MRI) studies suggests that the caudate nucleus (CN) plays a role in the cognitive, sensory, and emotional integration of pain information in patients with migraine. However, evidence concerning the role played by CN in CM patients is limited. Here, we used the CN as the seed to explore patterns of functional connectivity (FC) among healthy controls (HCs), patients with episodic migraine (EM), and patients with CM.

**Methods:**

We included 25 HCs, 23 EM patients, and 46 CM patients in this study. All participants underwent resting-state functional MRI scans on a GE 3.0T MRI system. We performed seed-based FC analyses among the three groups using the bilateral CNs as seeds. We also compared the subgroups of CM (with and without medication overuse headache, males and females) and performed Pearson’s correlation analyses between FC values and the clinical features of CM patients.

**Results:**

FC values between the right CN and five clusters (mainly involved in emotion, cognition, and sensory-related brain regions) were higher in CM patients than in HCs. Compared to EM patients, enhanced FC values between the bilateral precuneus, left anterior cingulate gyrus, right middle cingulate cortex, right lingual gyrus, and right CN were shown in the CM patients. There were no significant differences between CM patients with and without MOH, males and females. FC values between the bilateral calcarine cortex, lingual gyrus, and right CN were positively correlated with body mass index. Moreover, right CN-related FC values in the left calcarine cortex and right lingual gyrus were inversely correlated with visual analogue scale scores for headaches.

**Conclusion:**

Our results revealed abnormal right CN-based FC values in CM patients, suggesting dysfunction of brain networks associated with pain perception and multi-regulation (emotion, cognition, and sensory). Aberrant FC of the CN can provide potential neuroimaging markers for the diagnosis and treatment of CM.

**Supplementary Information:**

The online version contains supplementary material available at 10.1186/s10194-022-01506-9.

## Background

Migraine is a disabling neurological disorder with a worldwide prevalence of 14% (males, 8.6%; females, 17.0%) [1]. Chronic migraine (CM) affects approximately 1–2% of the global population [2], and patients with episodic migraine (EM) progress to CM at a rate of 2.5% per year [3]. Patients with CM have reduced household productivity, more disabilities, and worse quality of life relative than those with EM [4]. CM is defined as headaches on at least 15 days (migraine-like attacks ≥ 8 days) per month for more than 3 consecutive months [5]. Medication overuse is regarded as a consequence of chronic headache, a condition that is particularly common among migraineurs and tends to form medication overuse headache (MOH) [6]. A growing number of researchers now believe that MOH is a sequel or complication to CM rather than a separate entity [7, 8]. In recent years, approximately half of all CM patients have been considered comorbid with MOH—a condition that further complicates the clinical course of CM and its treatment [9]. However, the definitive pathophysiological mechanisms responsible for CM are not well understood.

In past decades, advanced neuroimaging technologies have been used to study the pathophysiology of migraine and explore its potential neuroimaging markers. Several studies have used functional and structural magnetic resonance imaging (MRI) to show that CM is closely associated with changes in the intrinsic brain network [10–14]. A study used brain functional connectivity (FC) to compare CM and EM and revealed that CM patients have stronger FC within the pain matrix [15]. CM is also associated with cognitive–emotional dysfunction. The caudate nucleus (CN) is a pivotal part of the basal ganglia (BG), which has been reported to be involved in the cognitive, sensory, and emotional integration of pain input [16]. Based on existing knowledge, the CN appears to be associated with migraine attacks [17] and chronification [18]. One study reported that caudate volumes were larger in migraine patients and were positively correlated with attack frequency [17]; however, another study reported reduced left caudate volumes in migraine patients [19]. Previous functional MRI (fMRI) studies on amplitude of low-frequency fluctuations, regional homogeneity, and functional connectivity density (FCD) have discovered abnormalities in the CN of migraine and MOH populations compared with those in the control population [20–22]. Enhanced FC between the hypothalamus and the CN has also been reported in individuals with CM [23]. One study compared the bilateral temporal pole, ipsilateral insula, ipsilateral middle frontal gyrus, and contralateral parahippocampus of migraine patients and reported a significantly lower FC of the CN in patients whose migraine episodes progressed than in patients whose migraine episodes did not progress [24]. In contrast, another study reported significantly enhanced FC of the left CN in the bilateral parahippocampal gyrus, right amygdala, and right insula of patients with migraine compared to HCs [17].

Prior studies have not reached uniform conclusions regarding changes in the FC of the CN in patients with migraine. Moreover, there is little research on CM populations, and role of the CN in CM is unclear. In this study, we investigated the involvement of the CN in the pathophysiology of CM and divided the CM population into subgroups to explore whether combined MOH or gender difference impacts the FC. We hypothesized that the FC of the CN is disrupted in CM patients and that the combined MOH affects the FC. We also assessed whether the clinical characteristics of CM were correlated with FC.

## Methods

### Study population

We prospectively enrolled 105 participants, including 28 healthy controls (HCs), 27 patients with EM, and 50 patients with CM. The 77 participants with migraine were patients who presented at Beijing Tiantan Hospital (Capital Medical University) from October 2020 to July 2022. Each patient was diagnosed by at least two neurologists who specialized in headaches. The inclusion criteria were as follows: (1) All diagnoses of EM and CM (with and without MOH) fulfilled the International Classification of Headache Disorders 3rd edition (ICHD-3). All patients had migraine without aura. MOH is defined as a complication of CM that involves the frequent overuse of medication to treat acute headaches [6, 9, 25]. (2) No migraine prophylactic medication within the preceding 3 months. (3) Right-handed. (4) Volunteered to engage in the study. All participants underwent physical examinations, routine laboratory tests, and imaging examinations before enrollment. After enrollment, they completed a headache questionnaire that collected data regarding headache history, headache days per month, medication use, and some related scales in the preceding 3 months. All patients with migraine completed the Migraine Disability Assessment Scale (MIDAS), Headache Impact Test-6 (HIT-6), Patient Health Questionnaire-9 (PHQ-9), Generalized Anxiety Disorder-7 (GAD-7), Pittsburgh Sleep Quality Index (PSQI), and Visual Analogue Scale (VAS). Higher scores of the HIT-6, PHQ-9, GAD-7, and PSQI represent greater impact of headaches, more serious depression, anxiety and sleep disturbance respectively. The VAS is used to evaluate the intensity of headache pain. A 3T MRI was performed to collect imaging data, and participants were instructed to avoid alcohol, caffeine, nicotine, and other substances for a minimum of 12 h prior to the MRI examination.

The exclusion criteria were as follows: (1) Existence of any cardiovascular, metabolic, or psychiatric disorders; cranium trauma; other subtypes of headache; and chronic pain disorder that may result in the overuse of analgesics. (2) Use of substances (such as alcohol or nicotine) other than the drugs included in the diagnostic criteria. (3) Pregnancy or menstrual period in women; (4) Inability to tolerate MRI examinations (that is, conditions such as claustrophobia). (5) Poor quality of data from MRI scans. Healthy controls should meet all of the above inclusion criteria except for the first one and fufill all the exclusion criteria.

This cross-sectional study was a component of a clinical trial (NCT05334927). The institutional review board of Beijing Tiantan Hospital approved the study protocols and procedures. The study was approved by the Ethics Committee of Beijing Tiantan Hospital, Capital Medical University (approval no. KY2022-044). Before their inclusion in the study, all participants gave written informed consent under the principles of the Declaration of Helsinki.

### Image acquisition

All imaging was performed with a GE 3.0 T MRI system (Signa Premier, GE Healthcare) equipped with a 48-channel head coil at the National Neurological Centre in Beijing Tiantan Hospital. Foam padding and earplugs were used to minimize noise from head movements and the scanner. Each participant was required to keep their head and neck steady, remain awake, close their eyes, and not think about anything during the 330-second blood oxygen level-dependent (BOLD) signal scan. A high-resolution 3D T1-weighted anatomical image was collected using the MP-RAGE sequence with 1.0 × 1.0 × 1.0 mm^3^ resolution (preparation time = 880 ms, recovery time = 400 ms, acceleration factor = 2, acquisition time = 4:00, field of view = 250 × 250 mm^2^, flip angle = 8°, axial slices = 192). Resting-state BOLD functional images were obtained using a multi-band BOLD sequence with 2.4 × 2.4 × 2.4 mm^3^ voxel size. The following fMRI parameters were used: transverse acquisition, slice number = 65 with multi-band factor = 6, flip angle = 64°, TE/TR = 39/1000 ns, no in-plane acceleration or slice gap, time point = 330, field of view = 208 mm, acquisition matrix = 86.

### Pre-processing of fMRI data

All image data were analyzed using the SPM12 (fil.ion.ucl.ac.uk/spm) and Data Processing Assistant for Resting-State fMRI (DPARSF, advanced edition) (http://www.rfmri.org/DPARSF) tools in MATLAB (Mathworks, Natick, MA, USA). Data pre-processing in SPM12 was conducted as follows: (1) removal of the first twenty volumes of each functional time course; (2) slice-time correction; (3) realignment; (4) co-registration; (5) spatial normalization; and (6) data smoothing with an 8 mm FWHM Gaussian kernel. To minimize artifacts, we excluded data in which we detected a displacement of > 1.5 mm in any direction or a head rotation angle > 1.5°. Structural images were partitioned into maps of gray matter, white matter, and cerebrospinal fluid, and the scans were normalized to the Montreal Neurological Institute brain template. The effects of low-frequency drift and high-frequency noise were removed using linear trend removal and temporal bandpass filtering (0.01–0.08 Hz) in DPARSFA.

### Seed-based analysis of FC

Seed-to-whole-brain FC data were obtained as described henceforth. The bilateral CNs were used as the seeds, which were defined based on the automated anatomical labelling (AAL) atlas [26]. The mean BOLD time course of voxels within the right and left CNs were extracted. Pearson’s correlation coefficients were calculated between the extracted time course and the time courses of the entire brain in a voxel-wise manner. The individual r-scores were transformed to normally distributed Z-scores with Fisher’s z transformation, and these were used in a general linear model analysis. The value of each voxel throughout the entire brain was considered representative of the degree of relative FC to each seed. Z-maps were subsequently used to analyze relative FC using SPM12.

In our analysis, we determined the differences in whole-brain resting-state FC in each CN seed region among the three groups. First, we performed one-way analysis of variance (ANOVA) with age and gender as covariates. Next, we performed a two-sample t-test at the level of the voxel with gender and age as covariates. Group-level effects were deemed significant if they were below the uncorrected voxel-level threshold of *p* < 0.001 (two-tailed) and the family-wise error-corrected cluster extent threshold of *p* < 0.05. Finally, the mean FC values (Z-scores) for the remaining clusters of CM were extracted and used for Pearson’s correlation analysis against clinical data.

### Statistical analysis

All data were presented as the mean ± standard deviation (SD). The chi-square test was used to compare differences between genders. Kruskal-Wallis H test was performed to compare continuous variables but nonnormal data such as age and body mass index (BMI) among three groups. Two sample t-test was applied to compare normally distributed data (PSQI scores) between the EM and CM groups. Mann-Whitney U tests were conducted to compare the non-normally distributed data (duration of migraine history, headache frequency, VAS scores, MIDAS scores, HIT-6 scores, PHQ-9 scores and GAD-7 scores) of patients the between EM and CM groups. Correlations between clinical characteristics and CN-based FC values (Z-scores) were analyzed using Pearson’s correlation analysis. Positive and negative correlations were represented by positive and negative correlation coefficients (r), respectively. The level of statistical significance was set at *p* < 0.05. All statistical tests were two-tailed and were analyzed using SPSS statistical software (version 26.0; IBM Corp., Armonk, NY, USA).

## Results

### Demographics and clinical data

**Fig. 1:**
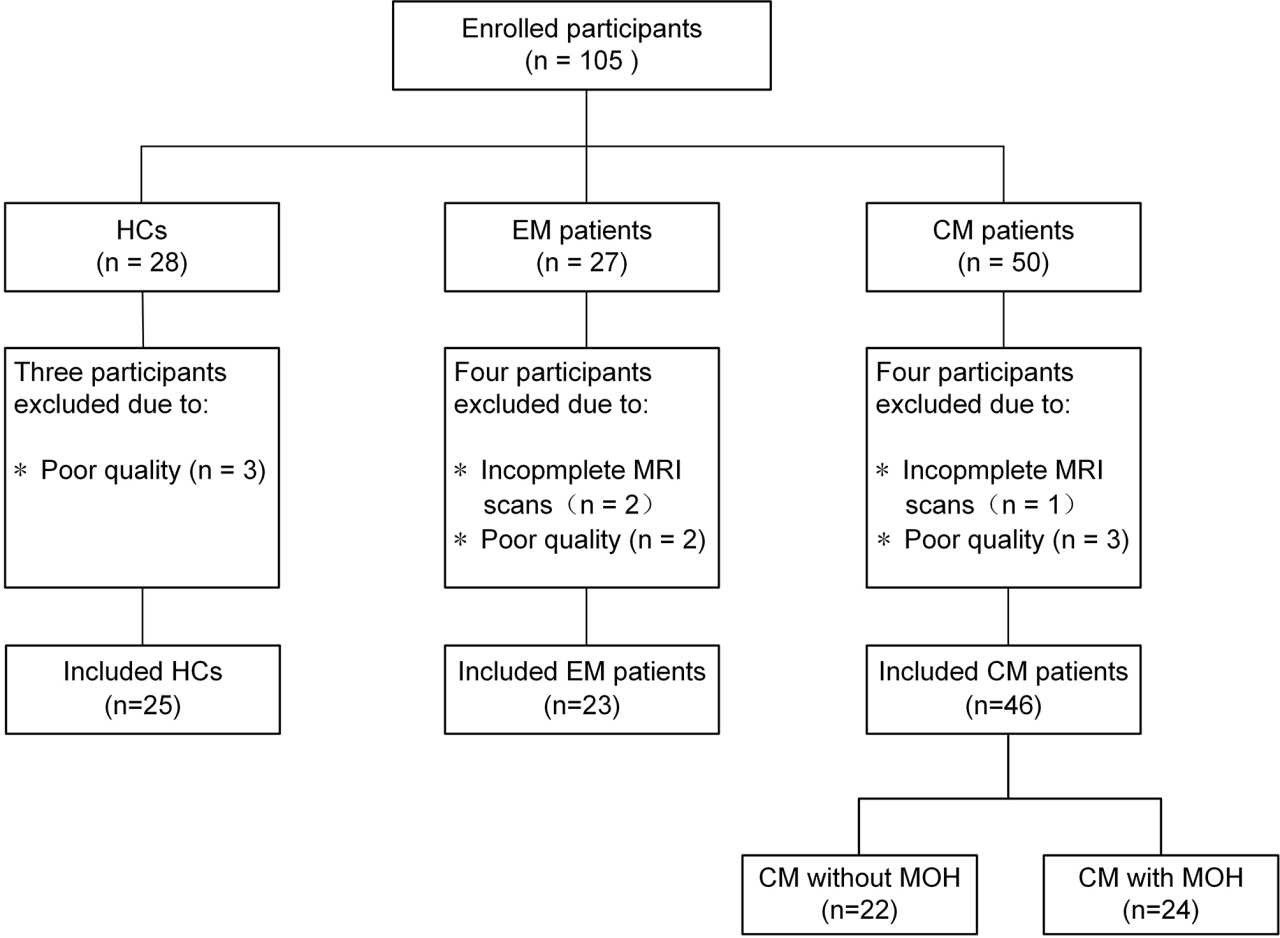
**Flowchart of the participant inclusion process** Note: CM, chronic migraine; EM, episodic migraine; MOH, medication overuse headache

The demographic and clinical features of enrolled participants in each group are listed in Table 1. Three HC participants were excluded because of poor MRI data quality; four EM patients were excluded owing to incomplete MRI scans (n = 2) and poor quality of data (n = 2), and four CM patients were excluded owing to incomplete MRI scans (n = 1) and poor quality of data (n = 3) (Fig. 1). We finally included 87 participants, including 25 HCs, 23 patients with EM, and 46 patients with CM (22 without MOH and 24 with MOH). The results of the chi-square test and Kruskal-Wallis H test revealed no significant differences among groups (EM group, CM group, and HCs) in terms of gender (*p* = 0.510), age (*p* = 0.426), and BMI (*p* = 0.205) (Table 1). For CM patients, the mean duration of migraine history was 17.70 ± 12.17 years, with a mean of 24.11 ± 6.27 headache days per month. EM patients had a mean duration of migraine history of 12.90 ± 9.79 years, with a mean of 8.09 ± 6.52 headache days per month. The MIDAS (*p* < 0.001), PHQ-9 (*p* < 0.001) and GAD-7 (*p* = 0.006) scores were significantly higher in CM patients than that in EM patients. However, the VAS (*p* = 0.288), HIT-6 (*p* = 0.315) and PSQI (*p* = 0.390) scores were not significantly different between the CM and EM groups. Within the subgroups of CM, CM patients with MOH had a longer duration of EM history (*p* = 0.021), longer duration of CM history (*p* < 0.001), and higher headache frequency (*p* = 0.033) than CM patients without MOH (Fig S1, Table 2). There were no significant differences in other characteristics between these groups. Females of CM patients had a higher MIDAS scores (p = 0.027) than males (Table S1), with no differences in other clinical data.

**Table 1:** Demographic and clinical data

	Controls (N = 25)	EM (N = 23)	CM (N = 46)	P value
Gender(female/male)	10 / 15	9 / 14	13 / 33	0.510^a^
Age(years)	35.68 ± 9.53	38.09 ± 11.56	38.80 ± 12.99	0.426^b^
BMI (kg/m^2^)	22.10 ± 3.24	24.40 ± 4.44	22.83 ± 3.55	0.205^b^
Migraine history (years)	-	12.90 ± 9.79	17.70 ± 12.17	0.131^c^
Headache frequency (d/mo)	-	8.09 ± 6.52	24.11 ± 6.27	<0.001^c^
Pain intensity VAS score	-	6.30 ± 2.08	6.96 ± 1.49	0.288^c^
MIDAS score	-	45.05 ± 32.61	125.70 ± 66.85	<0.001^c^
HIT-6 score	-	64.45 ± 7.37	65.93 ± 7.88	0.315^c^
PHQ-9 score	-	4.59 ± 4.03	10.78 ± 6.23	<0.001^c^
GAD-7 score	-	4.18 ± 4.68	7.98 ± 5.69	0.006^c^
PSQI score	-	8.85 ± 4.23	9.93 ± 4.83	0.390^d^
Medication overuse headache	-	-	24 (52.17%)	-

Note: EM, episodic migraine; CM, chronic migraine; BMI, Body Mass Index; d/mo, days per month; VAS, Visual Analogue Scale; MIDAS, Migraine Disability Assessment Scale; HIT-6, Headache Impact Test-6; PHQ-9, Patient Health Questionnaire-9; GAD-7, Generalized Anxiety Disorder-7; PSQI, Pittsburgh Sleep Quality Index.

Values represent mean ± SD or n (% of total)

^a^ Chi-square test

^b^ Kruskal-Wallis H test

^c^ Mann-Whitney U test

^d^ Independent samples t test

**Table 2:** Comparisons of demographics and clinical characteristics between CM with and without MOH

	Without MOH (N = 22)	With MOH (N = 24)	P value
Gender (female/male)	6 / 22	7 / 24	0.915^a^
Age (years)	36.77 ± 16.06	40.67 ± 9.33	0.660^b^
BMI (kg/m^2^)	22.65 ± 2.94	22.99 ± 4.09	0.974^b^
Migraine history (years)	13.50 ± 11.76	21.54 ± 11.46	0.021^b^
CM history (months)	16.73 ± 19.22	59.75 ± 59.96	< 0.001^b^
Headache frequency (d/mo)	22.00 ± 6.67	26.04 ± 5.31	0.033^b^
Pain intensity VAS score	6.68 ± 1.67	7.21 ± 1.28	0.306^b^
MIDAS score	136.41 ± 61.81	115.88 ± 71.02	0.303^c^
HIT-6 score	65.68 ± 6.67	66.17 ± 8.98	0.559^b^
PHQ-9 score	11.95 ± 5.74	9.71 ± 6.59	0.226^c^
GAD-7 score	9.05 ± 5.58	6.96 ± 5.72	0.222^c^
PSQI score	10.14 ± 5.15	9.74 ± 4.60	0.786^c^

Note: MOH, medication overuse headache; BMI, Body Mass Index; CM, chronic migraine; d/mo, days per month; MIDAS, Migraine Disability Assessment Scale; HIT-6, Headache Impact Test-6; PHQ-9, Patient Health Questionnaire-9; GAD-7, Generalized Anxiety Disorder-7; PSQI. Pittsburgh   Sleep Quality Index; VAS, Visual Analogue Scale.

Values represent mean ± SD or n (% of total)

^a^ Chi-square test

^b^ Mann-Whitney U test

^c^ Independent samples t test

### Seed-based rs-FC analysis in the HC, EM, and CM groups

**Fig. 2:**
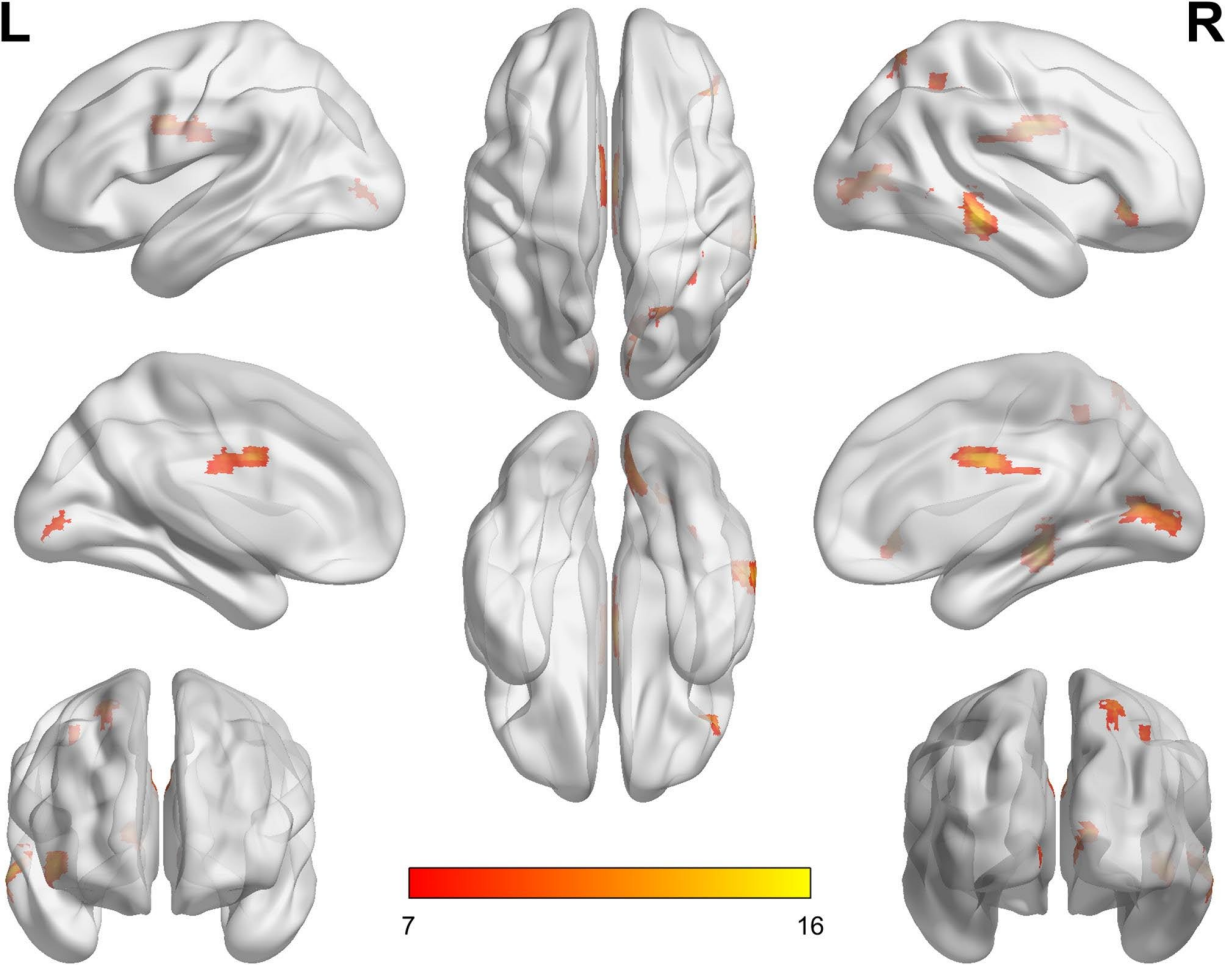
**Differences in the functional connectivity of the right caudate nucleus among the CM, EM, and HC groups.** The color bar represents the F-values Note: L, left; R, right; CM, chronic migraine; EM, episodic migraine; HC, healthy control

The bilateral CNs were chosen as the region of interests to explore the FC with the whole brain voxels. The results of an ANOVA among the HC, EM, and CM groups revealed that the FC of the right CN differed significantly in five clusters: the right middle temporal gyrus, bilateral calcarine cortex, right orbital part of the inferior frontal gyrus, bilateral middle cingulate cortex (MCC), and the right inferior and superior parietal cortex (Table 3; Fig. 2). There were no significant differences in the left CN.

**Table 3:** Brain regions showing seed-based resting-state FC differences of right CN between CM and EM, HCs

Brain regions	MNI coordinates			Peak *F/t* value	Cluster size	Cluster level P_FWE corr__._
	x	y	z			
**Three group comparison**						
Cluster 1	60	-33	-9	15.78	199	0.003
Temporal_Mid_R						
Cluster 2	12	-72	9	11.40	172	0.005
Calcarine_R						
Calcarine_L						
Cluster 3	42	33	-3	13.75	116	0.026
Frontal_Inf_Orb_R						
Frontal_Inf_Tri_R						
Cluster 4	3	-3	30	13.10	126	0.019
Cingulum_Mid_R						
Cluster 5	24	-66	63	14.12	169	0.006
Parietal_Sup_R						
Parietal_Inf_R						
**CM > HC**						
Cluster 1	12	-72	9	4.48	432	< 0.001
Calcarine_R						
Calcarine_L						
Lingual_R						
Lingual_L						
Cluster 2	27	-69	60	5.19	365	< 0.001
Parietal_Sup_R						
Parietal_Inf_R						
Precuneus_R						
Cluster 3	60	-30	-12	5.43	367	< 0.001
Temporal_Mid_R						
Temporal_Inf_R						
Temporal_Sup_R						
Cluster 4	42	33	-3	4.75	310	0.001
Insula_R						
Frontal_Inf_Orb_R						
Temporal_Pole_Sup_R						
Cluster 5	6	33	3	4.28	138	0.025
Cingulum_Ant_R						
Cingulum_Ant_L						
**CM > EM**						
Cluster 1	12	-30	36	4.62	182	0.012
Cingulum_Mid_R						
Precuneus_R						
Precuneus_L						
Cingulum_Ant_L						
Cluster 2	6	-42	3	4.51	124	0.043
Lingual_R						

Note: MNI, Montreal Neurological Institute; L, left; R, right; FWE, family-wise error corrected; CM, chronic migraine; EM, episodic migraine. Brain region localizations were performed using Automatic Anatomical Labeling (AAL) atlas and number of voxels of the anatomical regions in which the cluster extents to are reported. The surviving clusters were assigned thresholds at level p < 0.001 and FWE-corrected to p < 0.05 at the cluster level.

**Fig. 3:**
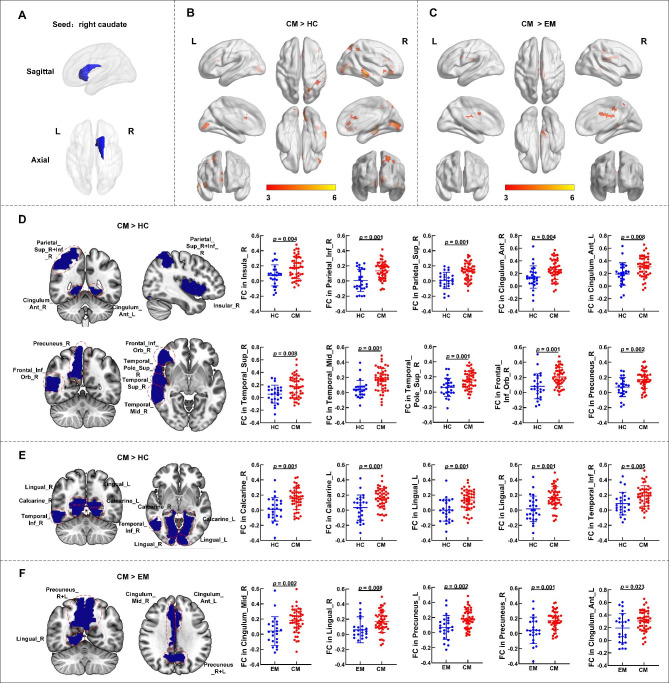
**Differences in the FC values of the right CN between the CM, EM, and HC groups.** (A). Right CN masks generated based on the automated anatomical labeling atlas. (B). When using the right CN as the seed, patients with CM show significantly higher FC values than HCs. (C). Patients with CM show significantly higher FC of the right CN than patients with EM. FC values in the (D).emotion-cognition related and (E). sensory-related brain regions show significant differences in HCs and patients with CM. (F). Patients with CM show significant FC values differences between the right CN and certain brain regions than EM patients. The color bar shows the t-values of the two-sample t-tests on FC. Note: L, left; R, right; FC, functional connectivity; CN, caudate nucleus; CM, chronic migraine; EM, episodic migraine; HC, healthy control.

Whole-brain FC analysis using the right CN as the seed revealed that compared with HCs, CM patients had higher FC values across a broad range of brain regions, including the bilateral calcarine cortex, bilateral lingual gyrus, bilateral anterior cingulate cortex (ACC), right superior parietal cortex, right inferior parietal cortex, right precuneus, right temporal lobe, right insula, right orbital part of the inferior frontal gyrus, and right temporal pole of the superior temporal gyrus (Fig. 3B; Table 3). FC values between the bilateral precuneus, left ACC, right MCC, and right CN were remarkably higher in the CM group than in the EM group (Fig. 3 C, Table 3). No significant differences were detected between HCs and the EM group.

### Seed-based rs-FC analysis between CM subgroups with and without MOH

There were large differences in clinical characteristics between the CM subgroups with and without MOH (Fig S1, Table 2). The results revealed no significant differences between CM patients with and without MOH.

### Seed-based rs-FC analysis between males and females of CM patients

Our results of CN-based FC suggested no significant differences between males and females in CM patients.

### Correlations between FC and clinical characteristics in CM patients

**Fig. 4:**
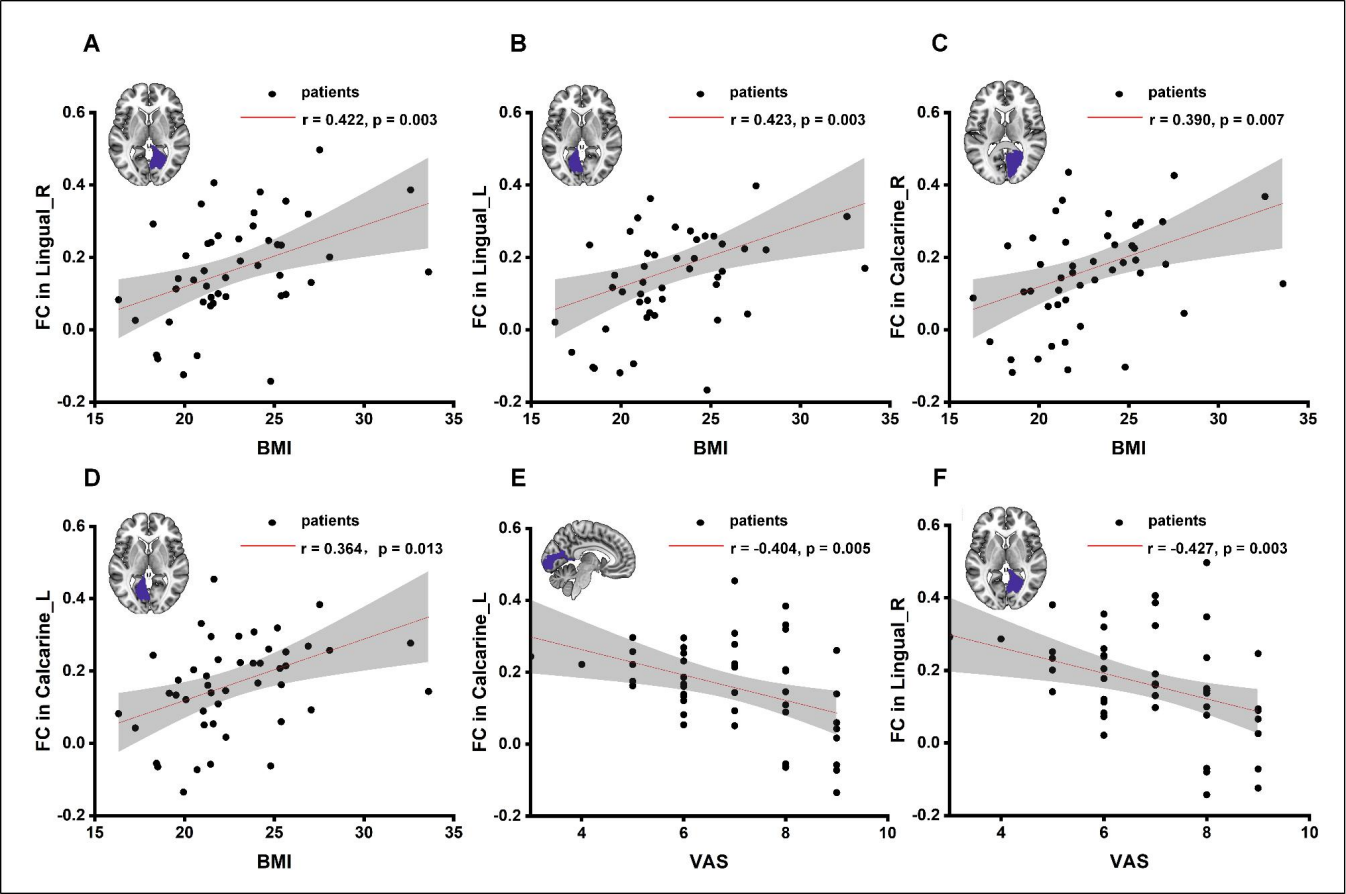
**Correlation between VAS scores, BMI, and FC of the right CN.** BMI is positively correlated with FC values between the right CN and the (A) Lingual_R (according to the AAL atlas); (B) Lingual_L; (C) Calcarine_R; and (D) Calcarine_L. VAS scores are negatively correlated with FC values between the right CN and the (E) Calcarine_L and (F) Lingual_R. Note: The results shown are from two-tailed tests (p < 0.05) for the significance of Pearson’s correlation coefficient. AAL, automated anatomical labeling; VAS, visual analogue scale; BMI, body mass index; L, left; R, right; FC, functional connectivity; CN, caudate nucleus.

For each brain region listed in Table 3, we explored the correlation between FC and the clinical characteristics of all patients with CM. The FC values between the right CN and left lingual gyrus were positively correlated with BMI (*p* = 0.003, r = 0.423, n = 46), and a similar pattern was noted for the right lingual gyrus (*p* = 0.003, r = 0.422, n = 46) (Fig. 4 A, B). Similarly, FC values between the right CN and bilateral calcarine cortex were positively correlated with BMI (left: *p* = 0.013, r = 0.364, n = 46; and right: *p* = 0.007, r = 0.390, n = 46) (Fig. 4 C, D). Furthermore, CN-related FC values in the left calcarine cortex (*p* = 0.005, r = -0.404, n=46) and right lingual gyrus (*p* = 0.003, r = -0.427, n = 46) were inversely correlated with VAS scores (Fig. 4E, F). There was no significant correlation between the FC values and other clinical characteristics (including the PHQ-9, GAD-7, HIT-6, PSQI, and MIDAS scores).

## Discussion

**Fig. 5:**
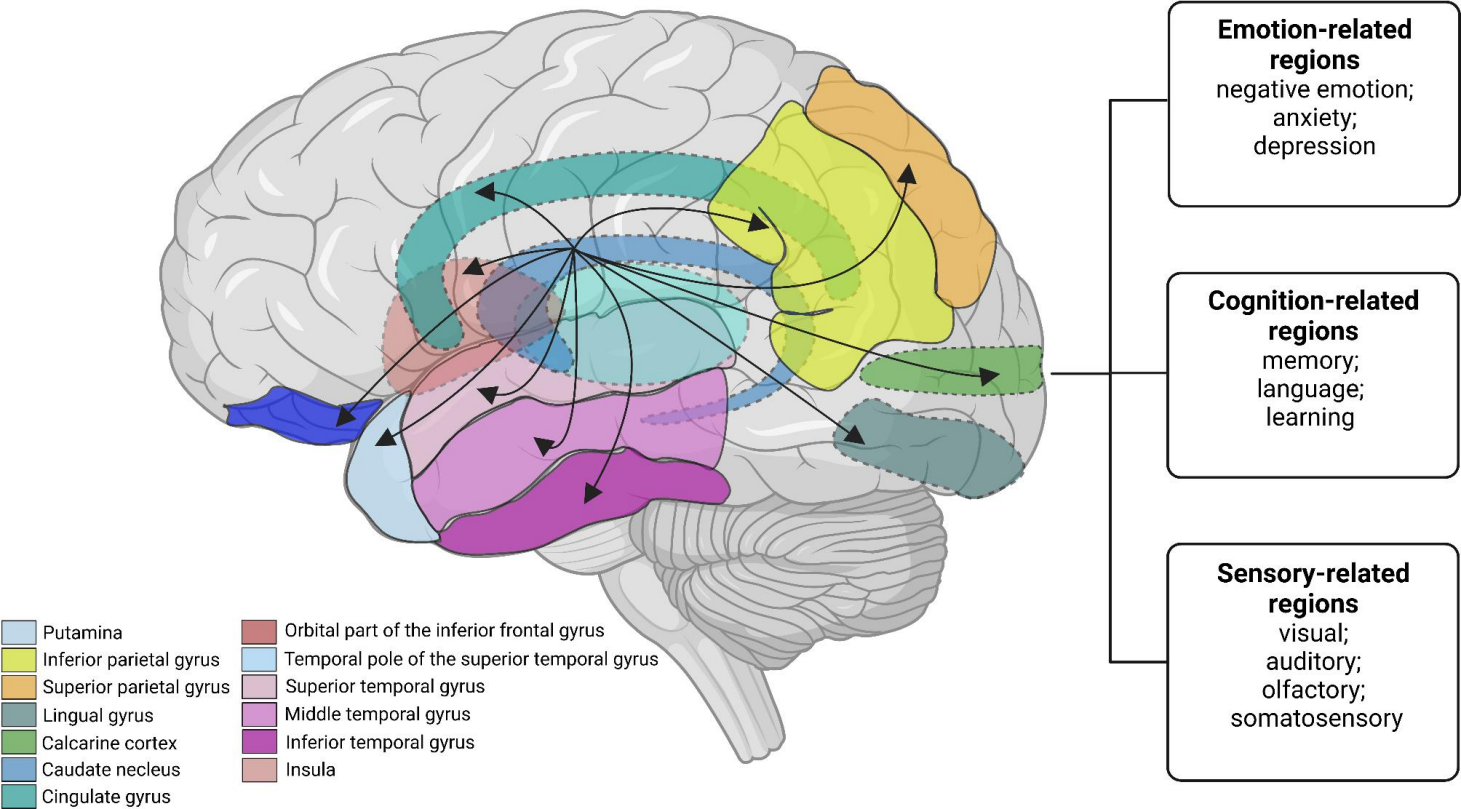
**Schematic diagram of the CM-related functional brain connectivity network.** The diagram shows our results about the significantly enhanced FC between the CN and brain regions associated with emotion, cognition, and sensory (the calcarine cortex, lingual gyrus, anterior cingulate cortex, superior and inferior parietal cortex, precuneus, temporal gyrus, insula, orbital part of the inferior frontal gyrus, and temporal pole of the superior temporal gyrus). The black arrows represent enhanced FC. The dotted line represents the tissue located inside the brain. Note: CM, chronic migraine; CN, caudate nucleus.

Our study firstly performed whole-brain seed-to-voxel analyses which focused on CN-based FC in CM patients compared with those in EM patients and HCs. The initial results revealed that patients with CM exhibited enhanced right CN-based FC with brain regions related to cognitive, emotional, and sensory functions (Fig. 5). Next, we found that the strength of right CN-based FC was correlated with clinical indicators of obesity (BMI) and headache intensity (VAS scores). Aberrant FC of these brain areas can contribute to the aberrant manifestations of CM such as memory loss, sensory abnormalities, cognitive impairment, cutaneous allodynia, sleep disorders, and increased susceptibility to migraine attacks. The dysfunction of the CN may be one of the pathophysiological mechanisms of CM. Nevertheless, as opposed to our assumptions, there were no significant differences among CM patients with and without MOH. This indicated that the aberrant FC in CM patients was not impacted by the combined MOH.

### The anatomical connections and functions of the CN

The CN is a deep cerebrum nucleus with C-shaped structure that consists of the head, body, and tail. It has a direct anatomical connection to the peripheral BG structures and exact fiber projection to the broad regions across the cerebral cortex. These connections engage in the formation of cortico-striato-thalamo-cortical circuits (CSTC) [27]. The striatum is an important part of the CSTC circuits, which consists of the CN and putamen and includes three parts (limbic, associative or cognitive, and sensorimotor areas). Each part creates a unique projection pattern by receiving projections from a different cerebral cortex [2]. The most ventral parts of the CN as one of the parts of the limbic area of the striatum, which mainly receives fibers from limbic and paralimbic cortices [28]. The associative or cognitive area has fiber connections with the frontal, parietal and temporal lobes, containing most of the CN [27, 29]. Similarly, the sensorimotor area receives the projection from the primary motor and somatosensory cortices, containing the dorsolateral edge of the head of the CN [27]. In addition, CN and putamen receive axons from almost all parts of the cortex excluding primary visual, auditory, and olfactory cortices [30]. The CN is involved in the regulation of emotion, cognition (memory, language, visuospatial, executive, computational, and comprehension judgments), movement, and sensation by connecting with these brain regions. Pain is a multidimensional composite with a highly specialized sensory experience [31]. Some evidence suggests an involvement of the CN in pain modulation by affecting the affective and cognitive processing of pain [32] and the CN might be regulated by the bioactive substance (arginine vasopressin, oxytocin, and so on) from the other brain regions, affecting the pain perception [33, 34]. Considering the CN has a complex and diverse function and the brain areas implicated in these projections correlate with the symptoms of CM, we selected the CN as a seed to explore whether it is involved in the pathophysiology of CM.

### Aberrant FC of CN in CSTC pathway associated with emotional-cognitive modulation

Cognitive–emotional dysfunction is prevalent in patients with CM, and emotions and different cognitive states (including the aspects of attention and memory) have been shown to modulate pain perception [3]. In our study, CM patients exhibited dysfunction in brain regions belonging to the CSTC pathway compared with HCs. In the CSTC circuits, the cortical axonal fiber projections to the striatum and enters the BG to the thalamus via direct and indirect pathways which recurrently terminates to the brain cortex [35], relating to emotional-cognitive modulation and pain processing.

The ACC and insula are mainly thought to be components of the limbic (emotional) system and are essential in coding the affective and motivational aspects of pain [36]. They are also a part of the somatosensory cortex, which perform the function of cognitive processing [37]. Our results confirmed that both the ACC and the insula affected the CN during pain perception, manifesting as enhanced FC between the CN and these brain regions. Prior findings concluded that the ACC and the CN formed an essential sequential pathway for processing pain avoidance behavior [38], which further implies the ACC and CN are associated with pain modulation. And the ACC is linked to the evaluation and expression of negative emotion [39], it has been found to show structural and functional changes in CM patients. CM patients tend to suffer from negative emotions or irritability when a headache occurs, and the negative emotional state often increases pain. This may explain why recurrent migraines are often combined with depression and anxiety disorders. People with CM have a 3.8 times higher risk of being depressed compared to HCs and approximately 37% of them have a psychiatric disorder [40]. The ventral CN with more interconnection to the limbic system, they are the part of the limbic CSTC that is implicated in affective functions [41]. This explains the mechanisms underlying our results, as the dysfunction of the limbic CSTC circuit could lead to abnormal clinical manifestations in CM. Nevertheless, other brain regions involved in this circuit did not show significant differences in our study. The other complex functions performed by these regions as well as the small sample size may explain it. The CSTC circuits also play a role in Parkinson’s disease and obsessive-compulsive disorders (OCD) [16, 42], and several studies have reported clinical observations regarding the interplay of migraine and the extrapyramidal system. Some findings have demonstrated that patients with BG disorders are more likely to suffer from migraines and suggested that these CSTC circuits can modify the course of migraine [16]. Taken together, these findings suggest that the dysfunction of this system not only leads to the occurrence of motor system disorders, but also chronification of migraine. Further studies need to focus on the role of this circuit in CM.

Previous studies have shown that CM patients with higher subjective and objective cognitive impairment mainly manifested the most striking deficits in memory/delayed recall (65.3%) [40]. In our study, CM patients showed enhanced FC between cognition-related brain regions and the right CN compared with HCs. These brain regions include the ACC, insular cortex, superior and inferior parietal gyrus, precuneus, and orbitofrontal cortex (OFC). These brain areas are jointly involved in cognitive regulation through projections to the CN and are also included in the cognition-related CSTC circuit. Damage to the CN has been known to cause cognitive impairment [43]. The parietal lobe was thought to have an association with the function of basic attention, language, and social cognition [44]. The precuneus belongs to the default mode network (DMN) that regulates higher-order cognitive function [14]. The OFC belonged to the frontostriatal circuits and importantly influences higher cognitive brain function [45]. A recent study has reported decreased intrinsic rs-FC in the cognitive networks such as the DMN, salience network, and central executive network (CEN) of CM patients [14]. In contrast, our results showed enhanced FC between the CN and cognition-related brain areas. Such inconsistent findings are likely due to the variations in sample recruitment, the proportion of gender, selection of seed, and the analysis methods. Our results suggest that the synchronous changes in BOLD signals between these brain regions may cause cognitive dysfunction in CM.

### Aberrant FC of CN in brain regions associated with sensory processing in CM

Our study found high FC values between the CN and brain regions related to sensory perception (including the calcarine cortex, lingual gyrus, temporal gyrus, temporal pole of the superior temporal gyrus, and inferior parietal gyrus) in CM patients compared to HCs. Previous MRI studies of CM patients have illustrated that corresponding brain networks with dysfunction primarily include the sensory networks (auditory network, AN; and visual network, VN). Enhanced FC in brain areas within the VN and AN implies an increased visual or auditory stimuli may potentiate the prominence of pain inputs, causing increased pain sensation [46]. In addition, neurons sensitive to visual, auditory, and somatosensory modalities have been identified in the CN and the lesions of CN can induce behavioral disturbances and over-reactivity, suggesting a failure of the inhibitory modulation of sensory inputs [36]. Visual signals transmitted through the retina are transmitted progressively along the occipito-parietal and occipito-temporal pathways [47]. The calcarine cortex and lingual gyrus as the pivotal areas for the visual pathway are associated with the perception of phosphenes [48], visual processing, and spatial memory [49]. Enhanced FC of the CN with the calcarine cortex and lingual gyrus has been presented among CM patients in our study, which may reflect disrupted visuomotor function and the failure of the CN in modulating sensory inputs. These were consistent with certain findings that impairments of visuomotor speed processing were observed in migraine patients with a higher frequency of headache attacks and a longer course of the disease [50]. The temporal pole is involved in comprehensive multisensory processing and integrates visual, auditory, olfactory, and somatosensory stimuli [51]. The region ranging from the inferior temporal visual cortex to the tail of the CN forms the visual corticostriatal loop that processes visual information and contributes to working memory and cognitive strategy selection [44]. As described in the diagnostic criteria, photophobia and phonophobia are two key non-head pain symptoms that are indicative of migraine [52]. The visual pathway and some related regions may lead to the symptoms of migraine. Therefore, our findings suggest that abnormal FC among the sensory-related regions reflects deficits in sensory processes in CM patients.

FC values between the CN and the ACC, MCC, lingual gyrus, and precuneus were also higher among CM patients than in EM patients. Patients with CM reported more somatic complaints—including fatigue, sleep disturbances, and nausea—compared with EM patients [53]. Moreover, CM patients are more susceptible to exhibiting comorbid psychiatric disorders, such as depression, anxiety, and OCD [40]. Our results provide further support for these conclusions. However, unlike in previous studies, we failed to find any marked differences in the CN-based FC between EM patients and HCs. This may be related to our seed selection. The seeds used in previous studies were mostly spherical regions in the CN. The neurons of these seeds were responsible for specific functional aspects in the EM population and showed significant differences compared with those in HCs. However, we used the entire CN based on the AAL template as the seed, representing the total function of the CN. Therefore, differences in CN-based FC values were only apparent between CM patients and HCs, not between EM patients and HCs. Similar patterns have been reported in a previous study showing that lower levels of GABA/Water and GABA/Cr were only observed in CM vs. HC comparisons, and not in EM vs. HC comparisons [25].

### CN-based FC in CM patients with and without MOH

Several imaging studies have reported specific brain changes in MOH patients that help differentiate MOH from other types of headaches [54]. However, an increasing number of researchers consider MOH to be a complication of other headaches as well [6–9]. CM patients with a high frequency of migraine attacks tend to overuse acute analgesics, which increases the risk of MOH. MOH may also contribute to the progression of CM. Our results indicated that the combined MOH may not lead to significant changes in CN-based FC in CM. An FCD study reported that compared with EM patients, MOH patients showed lower FCD in the right CN with a fronto-temporal-parietal distribution pattern [21]. The results of the FCD analysis suggested that the FC in the CN decreased in the MOH population without CM but increased in the EM population. In our study, the combined MOH was based on the CM diagnosis that means MOH developed from CM. We did not include the other primary headache and a subgroup of patients with MOH evolve from other headache types. Therefore, we can only conclude that MOH that developed from CM may not influence the FC of the CN. Some evidences showed that CM patients with MOH had improved FC between the prefrontal cortex and regions of the CN and frontal cortex within the CEN after treatment[55]. A magnetic resonance spectroscopy study suggested that there was no difference in neurochemical levels between CM patients with and without MOH [25]. Our findings further support that MOH is only comorbidity of CM and the presence of MOH would not change certain intrinsic properties of CM.

### Clinical characteristics of CM and correlations with FC values

Correlations between FC values of the CN and the characteristics of CM remain unclear. Our results showed that FC between the bilateral calcarine cortex, bilateral lingual gyrus, and CN were positively correlated with BMI. One study found that a higher BMI was associated with lower FC in the caudate-dorsolateral prefrontal cortex and higher FC in the caudate-medial temporal lobe [56]. The CN is a part of the reward system that is closely connected to BMI, and a higher BMI is considered to be associated with poorer cognitive flexibility. Previous studies have observed higher rs-FC values between the sensorimotor network and DMN in individuals with obesity [57]. Moreover, obesity is also a critical risk factor for migraine progression [58, 59]. Based on these findings, we speculate that the dysfunction of right CN-based FC with the visual cortex can damage the processing of visual information and cause abnormal sensory and cognitive impairments in CM patients, and these dysfunctions may be related to obesity. Susceptible populations with higher BMI scores may progress to CM earlier and exhibit more severe symptoms.

The VAS scores for headaches were inversely correlated with right CN-related FC in the left calcarine cortex and right lingual gyrus. The CN has been reported to play an essential role in sensory processing and pain inhibition [60]. The visual cortex can independently encode and differentiate visual cues related to pain anticipation [61]. And the pain in turn can alter visual cortex excitability that might reflect defensive strategies against pain [62]. Based on this, we believe that the functional network of the visual cortex and CN is involved in pain perception and the regulation of CM.

### Study limitations and future directions

This study has three principal limitations that need to be addressed in future studies. First, the sample size was relatively small, and the included population may not fully represent the characteristics of the entire patient population. We seek to enroll a larger population in future studies to further explore the mechanisms of CM. Second, we selected the bilateral CNs as the region of interests. The CN is an elongated C-shaped nucleus that consists of three subregions: the head, body, and tail. Different subregions of the CN may have unique functions; however, we have not discussed the FC of each part separately. Third, this was a cross-sectional study, and the results did not indicate any specific causal relationships between the altered FC of the CN and the formation of CM. In the future, we will conduct follow-up visits, connect better clinical information, and implement a better study design to address these limitations.

## Conclusion

We found evidence of multiple functional alterations in the brain networks of CM patients. These findings point toward a disruption in the pain modulatory system of CM patients (comprising emotional, cognitive, and sensory brain networks) and CSTC circuits that further our understanding of the clinical manifestation and pathogenesis of CM. MOH (a comorbidity of CM) and gender differences had no influence on the FC of the CN. Correlations between the FC and clinical data suggested that the CN was an important brain area that might have a certain pattern of interaction with the BMI and headache intensity of CM patients through a series of intrinsic brain networks. Above all, aberrant FC of the CN can provide potential neuroimaging markers for diagnosis and treatment of CM.

## Electronic supplementary material

Below is the link to the electronic supplementary material.

Supplementary Material 1

Supplementary Material 2

## Data Availability

Data can be made available upon request.

## Abbreviations

ACC: Anterior cingulate gyrus
AAL: Automated anatomical labelling
ANOVA: Analysis of variance
AN: Auditory network
BOLD: Blood oxygen level-dependent
BG: Basal ganglia
BMI: Body mass index
CM: Chronic migraine
CN: Caudate nucleus
CSTC: Cortico-striatal-thalamo-cortical
CEN: Central executive network
DMN: Default mode network
DPARSF: Data Processing Assistant for Resting-State fMRI
EM: Episodic migraine
FC: Functional connectivity
FCD: Functional connectivity density
fMRI: Functional magnetic resonance imaging
GAD-7: Generalized Anxiety Disorder-7
HC: Healthy control
HIT-6: Headache Impact Test-6
MOH: Medication overuse headache
MIDAS: Migraine Disability Assessment Scale
MCC: Middle cingulate gyrus
MRI: Magnetic resonance imaging
OCD: Obsessive-compulsive disorders
OFC: Orbitofrontal cortex
PSQI: Pittsburgh Sleep Quality Index
PHQ-9: Patient Health Questionnaire-9
SD: Standard deviation
VAS: Visual Analogue Scale
VN: Visual network

## References

[1] Stovner LJ, Hagen K, Linde M, Steiner TJ (2022). The global prevalence of headache: an update, with analysis of the influences of methodological factors on prevalence estimates. J Headache Pain.

[2] Natoli JL, Manack A, Dean B (2010). Global prevalence of chronic migraine: a systematic review. Cephalalgia: an international journal of headache.

[3] Bigal ME, Serrano D, Buse D, Scher A, Stewart WF, Lipton RB (2008). Acute migraine medications and evolution from episodic to chronic migraine: a longitudinal population-based study. Headache.

[4] May A, Schulte LH (2016). Chronic migraine: risk factors, mechanisms and treatment. Nat reviews Neurol.

[5] Headache Classification Committee of the International Headache Society (IHS) The International Classification of Headache Disorders, 3rd edition. Cephalalgia: an international journal of headache (2018) ;38:1-211 10.1177/0333102417738202 29368949

[6] Takahashi TT, Ornello R, Quatrosi G (2021). Medication overuse and drug addiction: a narrative review from addiction perspective. J Headache Pain.

[7] Diener HC, Dodick D, Evers S (2019). Pathophysiology, prevention, and treatment of medication overuse headache. Lancet Neurol.

[8] Martelletti P (2018). The journey from genetic predisposition to medication overuse headache to its acquisition as sequela of chronic migraine. J Headache Pain.

[9] Vandenbussche N, Laterza D, Lisicki M (2018). Medication-overuse headache: a widely recognized entity amidst ongoing debate. J Headache Pain.

[10] Planchuelo-Gómez Á, García-Azorín D, Guerrero ÁL, Aja-Fernández S, Rodríguez M, de Luis-García R (2020). White matter changes in chronic and episodic migraine: a diffusion tensor imaging study. J Headache Pain.

[11] Dai W, Qiu E, Chen Y (2021). Enhanced functional connectivity between habenula and salience network in medication-overuse headache complicating chronic migraine positions it within the addiction disorders: an ICA-based resting-state fMRI study. J Headache Pain.

[12] Coppola G, Di Renzo A, Petolicchio B (2019). Aberrant interactions of cortical networks in chronic migraine: A resting-state fMRI study. Neurology.

[13] Coppola G, Di Renzo A, Petolicchio B (2020). Increased neural connectivity between the hypothalamus and cortical resting-state functional networks in chronic migraine. J Neurol.

[14] Androulakis XM, Krebs K, Peterlin BL (2017). Modulation of intrinsic resting-state fMRI networks in women with chronic migraine. Neurology.

[15] Lee MJ, Park BY, Cho S, Kim ST, Park H, Chung CS (2019). Increased connectivity of pain matrix in chronic migraine: a resting-state functional MRI study. J Headache Pain.

[16] d’Onofrio F, Barbanti P, Petretta V (2012). Migraine and movement disorders. Neurol sciences: official J Italian Neurol Soc Italian Soc Clin Neurophysiol.

[17] Aldemir A, Yucel K, Güven H (2020). Structural neuroimaging findings in migraine patients with restless legs syndrome. Neuroradiology.

[18] Maleki N, Becerra L, Nutile L (2011). Migraine attacks the Basal Ganglia. Mol Pain.

[19] Yuan K, Zhao L, Cheng P (2013). Altered structure and resting-state functional connectivity of the basal ganglia in migraine patients without aura. J pain.

[20] Yu D, Yuan K, Zhao L, Liang F, Qin W (2013). Regional homogeneity abnormalities affected by depressive symptoms in migraine patients without aura: a resting state study. PLoS ONE.

[21] Chen Z, Chen X, Liu M, Dong Z, Ma L, Yu S (2017). Altered functional connectivity architecture of the brain in medication overuse headache using resting state fMRI. J Headache Pain.

[22] Li Z, Zeng F, Yin T (2017). Acupuncture modulates the abnormal brainstem activity in migraine without aura patients. NeuroImage Clin.

[23] Lerebours F, Boulanouar K, Barège M (2019). Functional connectivity of hypothalamus in chronic migraine with medication overuse. Cephalalgia: an international journal of headache.

[24] Seghatoleslam M, Ghadiri MK, Ghaffarian N, Speckmann EJ, Gorji A (2014). Cortical spreading depression modulates the caudate nucleus activity. Neuroscience.

[25] Wang W, Zhang X, Bai X (2022). Gamma-aminobutyric acid and glutamate/glutamine levels in the dentate nucleus and periaqueductal gray with episodic and chronic migraine: a proton magnetic resonance spectroscopy study. J Headache Pain.

[26] Tzourio-Mazoyer N, Landeau B, Papathanassiou D (2002). Automated anatomical labeling of activations in SPM using a macroscopic anatomical parcellation of the MNI MRI single-subject brain. NeuroImage.

[27] Çırak M, Yağmurlu K, Kearns KN (2020). The Caudate Nucleus: Its Connections, Surgical Implications, and Related Complications. World Neurosurg.

[28] Alheid GF, Heimer L (1988). New perspectives in basal forebrain organization of special relevance for neuropsychiatric disorders: the striatopallidal, amygdaloid, and corticopetal components of substantia innominata. Neuroscience.

[29] Goldman PS, Nauta WJ (1977). An intricately patterned prefronto-caudate projection in the rhesus monkey. J Comp Neurol.

[30] Grahn JA, Parkinson JA, Owen AM (2008). The cognitive functions of the caudate nucleus. Prog Neurobiol.

[31] Kuner R, Kuner T (2021). Cellular Circuits in the Brain and Their Modulation in Acute and Chronic Pain. Physiol Rev.

[32] Borsook D, Upadhyay J, Chudler EH, Becerra L (2010). A key role of the basal ganglia in pain and analgesia–insights gained through human functional imaging. Mol Pain.

[33] Pan YJ, Wang DX, Yang J (2016). Oxytocin in hypothalamic supraoptic nucleus is transferred to the caudate nucleus to influence pain modulation. Neuropeptides.

[34] Yang J, Li P, Zhang XY (2011). Arginine vasopressin in hypothalamic paraventricular nucleus is transferred to the caudate nucleus to participate in pain modulation. Peptides.

[35] Seger CA (2013). The visual corticostriatal loop through the tail of the caudate: circuitry and function. Front Syst Neurosci.

[36] Nagy A, Eördegh G, Paróczy Z, Márkus Z, Benedek G (2006). Multisensory integration in the basal ganglia. Eur J Neurosci.

[37] Emmert K, Breimhorst M, Bauermann T, Birklein F, Van De Ville D, Haller S (2014). Comparison of anterior cingulate vs. insular cortex as targets for real-time fMRI regulation during pain stimulation. Front Behav Neurosci.

[38] Koyama T, Kato K, Mikami A (2000). During pain-avoidance neurons activated in the macaque anterior cingulate and caudate. Neurosci Lett.

[39] Etkin A, Egner T, Kalisch R (2011). Emotional processing in anterior cingulate and medial prefrontal cortex. Trends Cogn Sci.

[40] Latysheva N, Filatova E, Osipova D, Danilov AB (2020). Cognitive impairment in chronic migraine: a cross-sectional study in a clinic-based sample. Arq Neuropsiquiatr.

[41] Huang H, Nguyen PT, Schwab NA, Tanner JJ, Price CC, Ding M (2017). Mapping Dorsal and Ventral Caudate in Older Adults: Method and Validation. Front Aging Neurosci.

[42] Brennan BP, Rauch SL, Jensen JE, Pope HG (2013). Jr. A critical review of magnetic resonance spectroscopy studies of obsessive-compulsive disorder. Biol Psychiatry.

[43] Villablanca JR (2010). Why do we have a caudate nucleus?. Acta Neurobiol Exp.

[44] Numssen O, Bzdok D, Hartwigsen G(2021) Functional specialization within the inferior parietal lobes across cognitive domains.eLife. ; 10 10.7554/eLife.63591 PMC7946436 33650486

[45] Levitt JJ, Zhang F, Vangel M, *et al*(1991) The Organization of Frontostriatal Brain Wiring in Healthy Subjects Using a Novel Diffusion Imaging Fiber Cluster Analysis. *Cerebral cortex (New York, NY*: 2021;31:5308–5318 10.1093/cercor/bhab159 PMC8568004 34180506

[46] Zou Y, Tang W, Qiao X, Li J (2021). Aberrant modulations of static functional connectivity and dynamic functional network connectivity in chronic migraine. Quant imaging Med Surg.

[47] Wen Z, Zhou FQ, Huang X, Dan HD, Xie BJ, Shen Y (2018). Altered functional connectivity of primary visual cortex in late blindness. Neuropsychiatr Dis Treat.

[48] Baier B, de Haan B, Mueller N (2010). Anatomical correlate of positive spontaneous visual phenomena: a voxelwise lesion study. Neurology.

[49] Sulpizio V, Committeri G, Lambrey S, Berthoz A, Galati G (2013). Selective role of lingual/parahippocampal gyrus and retrosplenial complex in spatial memory across viewpoint changes relative to the environmental reference frame. Behav Brain Res.

[50] Calandre EP, Bembibre J, Arnedo ML, Becerra D (2002). Cognitive disturbances and regional cerebral blood flow abnormalities in migraine patients: their relationship with the clinical manifestations of the illness. Cephalalgia: an international journal of headache.

[51] Schwedt TJ, Chong CD, Wu T, Gaw N, Fu Y, Li J (2015). Accurate Classification of Chronic Migraine via Brain Magnetic Resonance Imaging. Headache.

[52] Goadsby PJ, Holland PR, Martins-Oliveira M, Hoffmann J, Schankin C, Akerman S (2017). Pathophysiology of Migraine: A Disorder of Sensory Processing. Physiol Rev.

[53] Maizels M, Burchette R (2004). Somatic symptoms in headache patients: the influence of headache diagnosis, frequency, and comorbidity. Headache.

[54] Pozo-Rosich P, Coppola G, Pascual J, Schwedt TJ (2021). How does the brain change in chronic migraine? Developing disease biomarkers. Cephalalgia: an international journal of headache.

[55] Krebs K, Rorden C, Androulakis XM (2018). Resting State Functional Connectivity After Sphenopalatine Ganglion Blocks in Chronic Migraine With Medication Overuse Headache: A Pilot Longitudinal fMRI Study. Headache.

[56] Zhao J, Manza P, Gu J (2021). Contrasting dorsal caudate functional connectivity patterns between frontal and temporal cortex with BMI increase: link to cognitive flexibility. Int J Obes.

[57] Doucet GE, Rasgon N, McEwen BS, Micali N, Frangou S(1991) Elevated Body Mass Index is Associated with Increased Integration and Reduced Cohesion of Sensory-Driven and Internally Guided Resting-State Functional Brain Networks. *Cerebral cortex (New York, NY*: 2018;28:988–997 10.1093/cercor/bhx008 PMC6059200 28119342

[58] Bigal ME, Liberman JN, Lipton RB (2006). Obesity and migraine: a population study. Neurology.

[59] Cho SJ, Chu MK (2015). Risk factors of chronic daily headache or chronic migraine. Curr Pain Headache Rep.

[60] Freund W, Stuber G, Wunderlich AP, Schmitz B (2007). Cortical correlates of perception and suppression of electrically induced pain. Somatosens Motor Res.

[61] Machado AG, Gopalakrishnan R, Plow EB, Burgess RC, Mosher JC (2014). A magnetoencephalography study of visual processing of pain anticipation. J Neurophysiol.

[62] Coppola G, Serrao M, Currà A (2010). Tonic pain abolishes cortical habituation of visual evoked potentials in healthy subjects. J pain.

