# Supplementary material for: Altered functional connectivity of the right caudate nucleus in chronic migraine: a resting-state fMRI study
Source: J Headache Pain. 2022 Dec 2;23(1):154. doi: 10.1186/s10194-022-01506-9 (PMC9717534; doi:10.1186/s10194-022-01506-9)
Supplement: Supplementary file 1 — Supplementary Material 1 [file 10194_2022_1506_MOESM1_ESM.docx]

**Table S1 Comparisons of demographics and clinical characteristics between males and females in CM patients**

|  | Male  (N=13) | Female  (N=33) | P value |
| --- | --- | --- | --- |
| Age(years) | 36.69 ± 16.48 | 39.64 ± 11.52 | 0.714^b^ |
| BMI (kg/m^2^) | 23.52 ± 4.36 | 22.55 ± 3.21 | 0.400^b^ |
| Migraine history (years) | 19.31 ± 13.45 | 17.06 ± 11.79 | 0.565^b^ |
| CM history (months) | 51.31 ± 65.48 | 34.39 ± 42.42 | 0.374^b^ |
| Headache frequency (d/mo) | 22.15 ± 6.08 | 24.88 ± 6.26 | 0.184^b^ |
| Pain intensity VAS score | 6.77 ± 1.24 | 7.03 ± 1.59 | 0.462^b^ |
| MIDAS score | 91.31 ± 64.78 | 139.24 ± 63.57 | 0.027^c^ |
| HIT-6 score | 65.31 ± 10.44 | 66.18 ± 6.80 | 0.912^b^ |
| PHQ-9 score | 11.00 ± 6.43 | 10.70 ± 6.25 | 0.884^c^ |
| GAD-7 score | 8.15 ± 6.03 | 7.90 ± 5.64 | 0.896^c^ |
| PSQI score | 8.23 ± 5.36 | 10.63 ± 4.50 | 0.133^c^ |

Note**:** MOH, medication overuse headache; BMI, Body Mass Index; CM, chronic migraine; d/mo

days per month; MIDAS, Migraine Disability Assessment Scale; HIT-6, Headache Impact Test-6;

PHQ-9, Patient Health Questionnaire-9; GAD-7, Generalized Anxiety Disorder-7; PSQI,

Pittsburgh Sleep Quality Index; VAS, Visual analogue scale.

Values represent mean ± SD or n (% of total).

^a^ Chi-square test

^b^ Mann-Whitney U test

^c^ Independent samples t test
