# Supplementary figures and images for: Altered functional connectivity of the right caudate nucleus in chronic migraine: a resting-state fMRI study
Source: J Headache Pain. 2022 Dec 2;23(1):154. doi: 10.1186/s10194-022-01506-9 (PMC9717534; doi:10.1186/s10194-022-01506-9)

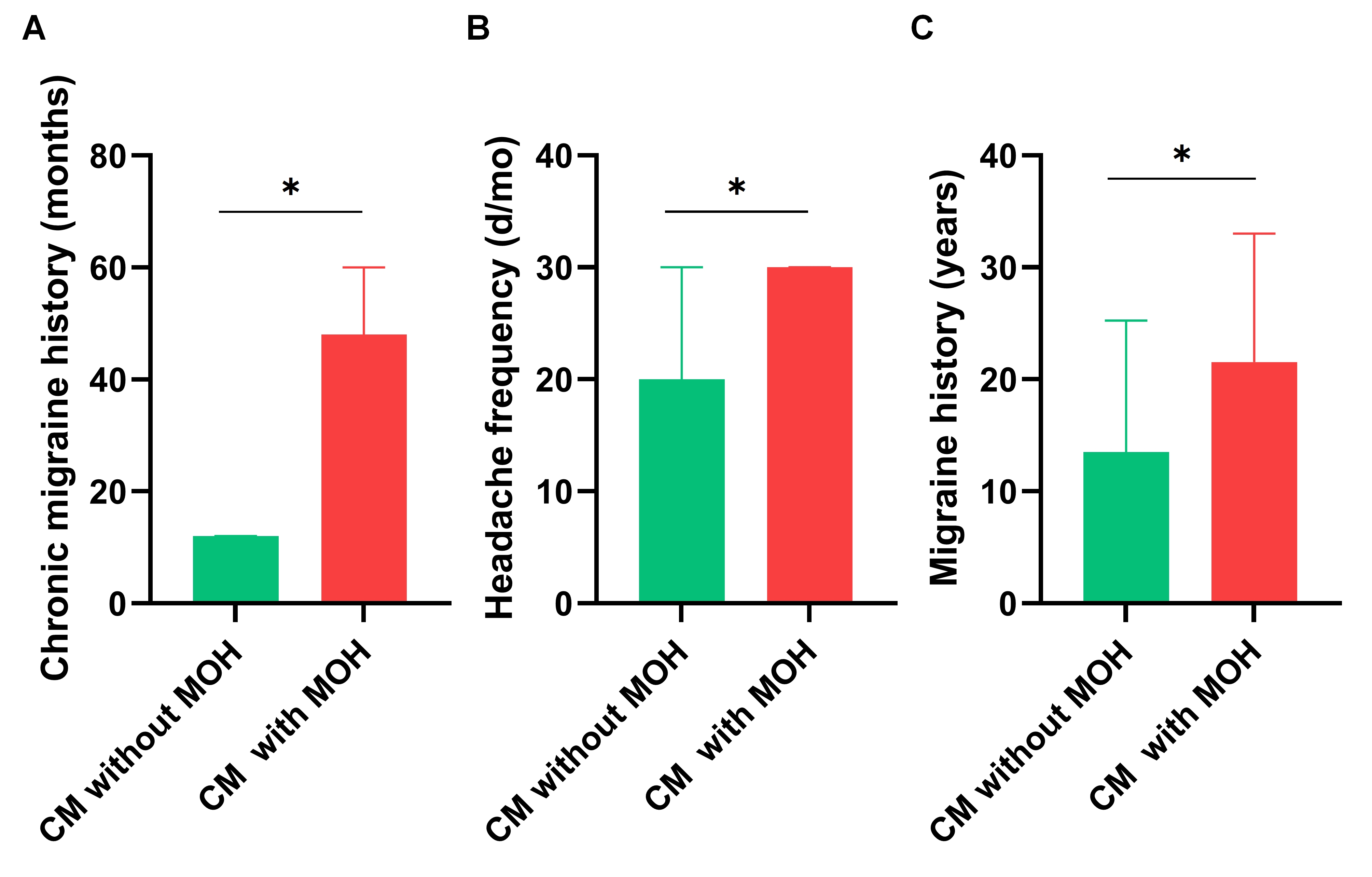

Supplement: Supplementary file 2 — Supplementary Material 2 [file 10194_2022_1506_MOESM2_ESM.jpg]
